# Impact of Donor and Recipient Single Nucleotide Polymorphisms of IL28B rs8099917 in Living Donor Liver Transplantation for Hepatitis C

**DOI:** 10.1371/journal.pone.0090462

**Published:** 2014-03-05

**Authors:** Nobuhiro Harada, Sumihito Tamura, Yasuhiko Sugawara, Junichi Togashi, Takeaki Ishizawa, Junichi Kaneko, Taku Aoki, Yoshihiro Sakamoto, Kiyoshi Hasegawa, Tomohiro Tanaka, Noriyo Yamashiki, Norihiro Kokudo

**Affiliations:** 1 Division of Artificial Organ and Transplantation, The University of Tokyo Hospital, Tokyo, Japan; 2 Division of Hepato-Biliary and Pancreatic Surgery, Department of Surgery, The University of Tokyo Hospital, Tokyo, Japan; 3 Organ Transplantation Service, The University of Tokyo Hospital, Tokyo, Japan; Saint Louis University, United States of America

## Abstract

Single nucleotide polymorphisms of interleukin-28B (IL28B) rs8099917 are reported to be associated with virologic clearance in interferon-and ribavirin -based treatment for hepatitis C virus (HCV)-infected patients. We examined virologic response in accordance with IL28B polymorphisms in our living donor liver transplantation series under a preemptive interferon and RBV treatment approach. Adequate DNA samples from both the recipient and donor for the study of single nucleotide polymorphisms of IL28B were available from 96 cases and were the subjects of the present study. Various clinical factors related with virologic response including early virologic response (EVR) and sustained virologic response (SVR) were examined. Totally 51% presented with EVR and 44% achieved SVR. Presence of the major allele (TT) in either the recipient or the donor corresponded to SVR of 53% and 48%. Presence of the minor allele (TG or GG) corresponded to SVR of 26% and 32%. Multivariate analysis revealed that genotype of HCV or EVR, but not IL28B polymorphisms in either the recipient or donor, was an independent factor for achieving SVR. When virologic response to treatment was incorporated into analysis, the impact of IL28B polymorphism on virological clearance remained relative to other factors and was not significantly independent.

## Introduction

Hepatitis C virus (HCV) infection is the leading cause of end-stage liver disease necessitating liver transplantation in developed countries [1]–[3]. HCV reinfection following liver transplantation is universal, however, and the histologic progression of HCV-related liver cirrhosis is accelerated in comparison with the non-transplant population [4]–[8]. Long-term outcomes are reported to be poorer in liver transplant recipients with HCV recurrence [9]–[10]. Although its efficacy remains unsatisfactory, the standard treatment for HCV relapse following liver transplantation is the combined application of pegylated interferon and ribavirin (RBV) [11]–[16]. To improve the overall outcomes of patients undergoing living donor liver transplantation (LDLT) for HCV-related liver disease, we routinely administered interferon based treatment preemptively in our series [17] with recent results indicating a sustained viral response (SVR) rate of 43% [18].

In a recent genome-wide association study, three independent institutions identified single nucleotide polymorphisms in the interleukin (IL)-28B gene on chromosome 19q13; rs12980275 or rs8099917 as being strongly associated with the virologic response to interferon and RBV-based treatment in HCV-infected patients [19]–[21]. In Japanese patients, the G nucleotide of rs8099917 (minor allele) is associated with a poor response to treatment, whereas a T nucleotide (major allele) is associated with a fair response [20].

The impact of the IL28B polymorphism was recently studied in the liver transplant setting. A small series reported that combined analysis of IL28B polymorphisms in both donors and recipients may predict the possibility of achieving SVR under current standard pegylated interferon-and RBV-based therapy [22]–[23]. The evidence, however, remains limited. In the present study, we examined the virologic response to a preemptive treatment approach in accordance with IL28B polymorphism.

## Patients and Methods

### Patients

From June 1996 to December 2012, 499 LDLT surgeries were performed at the University of Tokyo Hospital among which 134 cases were for HCV-related liver disease. Among them, three recipients remained negative for HCV RNA after transplantation alone, not requiring interferon treatment, and were excluded from the study. In the remaining 131, either appropriate informed consent or adequate samples for genetic analysis was not available in 25 cases for the following reasons and therefore excluded; death during the earlier period in 18, lost to follow-up in 3, and decline of study consent in 14 cases. The remaining 96 cases were the subjects of the present analysis. The earliest case of this population underwent LDLT in June 1998, and the latest case underwent LDLT in February 2011. Follow up was done until the end of 2012, or death.

### Antiviral therapy for HCV

Treatment was initiated with low-dose interferon alpha-2b and RBV 400 mg/day promptly after improvement of the general condition following liver transplantation. Recovery of hematologic and renal function was considered crucial. Specifically, initiation was considered when the leukocyte number > = 4000/ml, platelet count > = 50 000/ml, hemoglobin > =  8 g/l, and serum creatinine < 2 mg/dl. Thereafter, the dosage was gradually increased as tolerated. Finally, pegylated-interferon alpha-2b 1.5 µg/kg/week and RBV 800 mg/day were administered, depending on patient compliance [17], [18].

HCV RNA was measured quantitatively by reverse-transcriptase polymerase chain reaction (Amplicor HCV; Roche Molecular Systems, Pleasanton, CA). The response was considered to be an EVR at 12 week with an at least 2 log10 drop in serum HCV-RNA. The treatment was continued for 12 months after serum HCV-RNA turned negative, defined as ETR. The response was considered to be a SVR after another 6 months of negative serologic results without anti-viral treatment. Flexible dose adjustments were made as necessary to avoid serious adverse events and to prevent a lapse in treatment. A fixed overall treatment period length was not defined [17], [18].

### Analysis of the genotype of IL28B

Genomic DNA from recipients and donors was extracted either from peripheral blood mononuclear cells or from formalin-fixed paraffin-embedded liver biopsy samples depending on availability. Genotyping of single nucleotide polymorphisms IL28B rs8099917 was performed as previously described [20], [22].

### Ethics Statement

All LDLTs were performed after individually obtaining informed consent from recipients and donors. LDLT program at the University Of Tokyo Hospital has been approved by it's Institutional Review Board, and all aspects of the procedures have been conducted according to the principles expressed in the Declaration of Helsinki. The current human subject research was approved as project number G3514 by Graduate School of Medicine and Faculty of Medicine, the University of Tokyo Research Ethics Committee and Human Genome, Gene Analysis Research Ethics Committee. All subjects have been properly instructed and participated by signing the appropriate informed consent paperwork. In the preparation of this manuscript, all efforts have been made to protect patient privacy and anonymity.

### Statistical analysis

The cumulative rate of viral responses was studied using the Kaplan-Meier method. Comparison was made using the log-rank test. A multivariate analysis was performed using the Cox proportional-hazards model and a forward stepwise procedure. Categorical various clinical factors were compared between groups using the Fisher's exact test. JMP 9 software (SAS Institute Japan, Tokyo, Japan) was used for all analyses. A p-value of less than 0.05 was considered statistically significant.

## Results

### Patient demographics and virologic response

All recipients and donors of the 96 cases were of Japanese origin. Median age of the recipients and donors was 56 (range 23–66) and 34 (range 17–66), respectively. In 60 cases, the donor was a relative within a first degree of consanguinity. Among the recipients, 60 cases presented with hepatocellular carcinoma, 3 with HIV co-infection, and 2 with hepatitis B virus co-infection. The HCV RNA genotype was confirmed to be 1b in 79 (81%). Median calculated model for end-stage liver disease score at the time of transplant was 14.

All 96 recipients received interferon treatment according to our pre-emptive regimen described above. None presented with fibrosing cholestatic hepatitis at the time of treatment initiation. Overall, at the time of follow-up, 49 (51%) presented with early virologic response (EVR). Sixty-five (67%) patients had negative serum HCV RNA results at least once, among which 52 (54%) patients experienced a non-detectable level of serum HCV RNA for 12 months on treatment (end of treatment response, ETR). Forty-three (44%) patients remained negative for serum HCV RNA for 6 months or longer after cessation of interferon treatment following ETR, and these recipients were considered to have achieved SVR. Consistent with the nature of a treatment protocol without a defined time endpoint, the response rate increased over time. The cumulative rates of SVR and ETR based on the Kaplan-Meier method are 41% and 54%, respectively, 5 years after transplantation.

### Frequency of IL28B rs8099917 single nucleotide polymorphisms in recipients and donors

The proportion of TT, TG and GG genotypes in recipients was 72%, 28%, and 0%, respectively, whereas TT was the most frequent genotype in donors (80%), followed by genotypes TG (19%) and GG (1%). This distribution did not differ significantly between genotype 1b and the others.

### IL28B polymorphism and interferon sensitivity after LDLT

**Figure 1:**
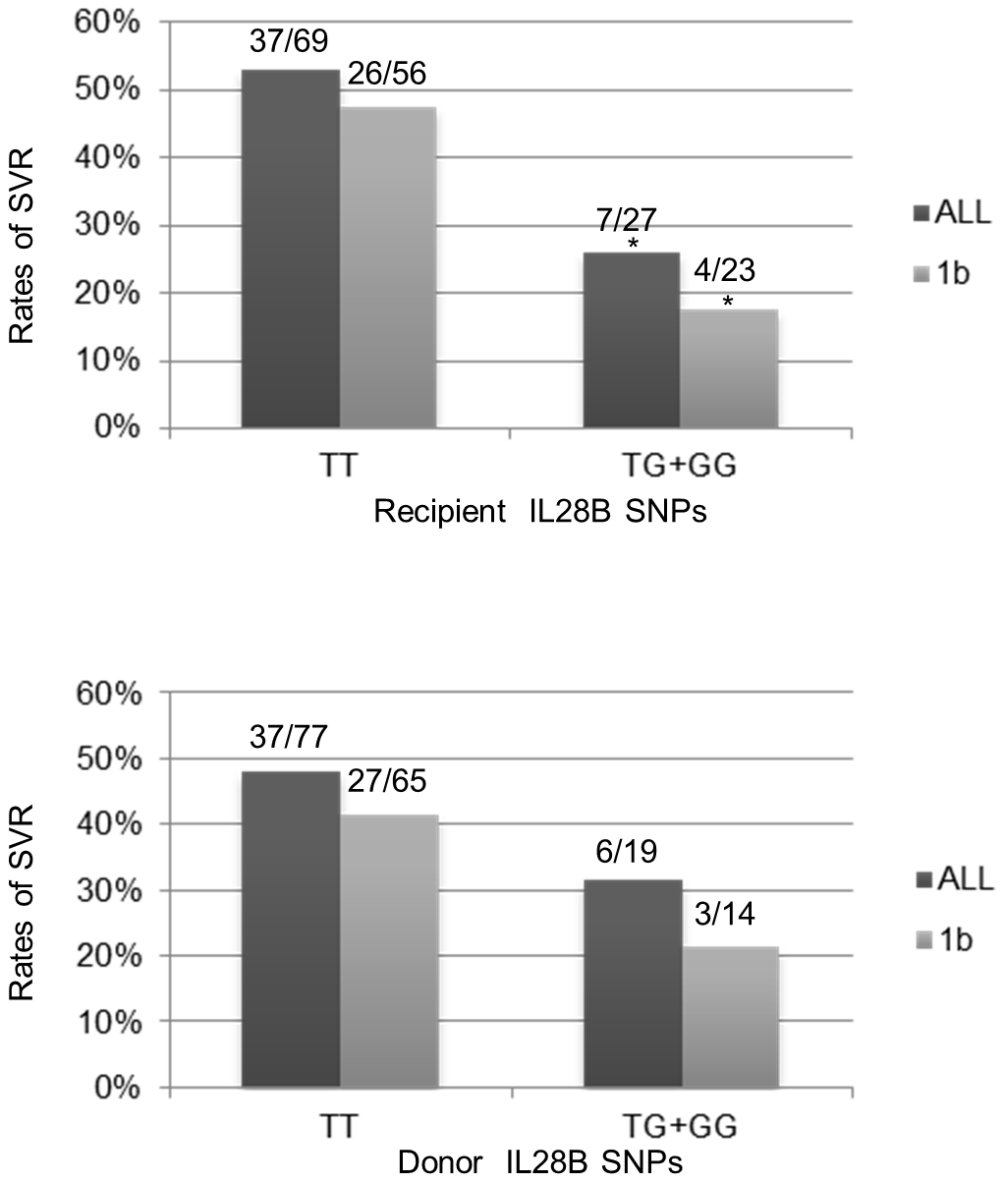
IL28B rs8099917 SNPs in the recipient and donor and corresponding rates of SVR. * indicates p = <0.05 v.s. major allele (TT) with corresponding background.

As previously reported, IL28B polymorphisms in both the recipient and donor seemed to be strongly correlated with the sensitivity to interferon treatment. The major allele (TT) in the recipient and donor corresponded to SVR rates of 54% (37 of 69) and 48% (37 of 77), respectively, whereas the presence of the minor allele (TG or GG) corresponded to SVR rates of 26% (7 of 27) and 32% (6 of 19), respectively (Figure 1). Difference of SVR between TT and TG/GG in recipient was statistically significant (p = 0.0219 and p = 0.0212 for all recipients and for recipients with genotype 1b HCV, respectively). However, difference of SVR according to the donor IL28B SNPs did not reach statistical significance (p = 0.3029 and p = 0.2279 for all donors and for donors whose recipients with genotype 1b, respectively).

**Figure 2:**
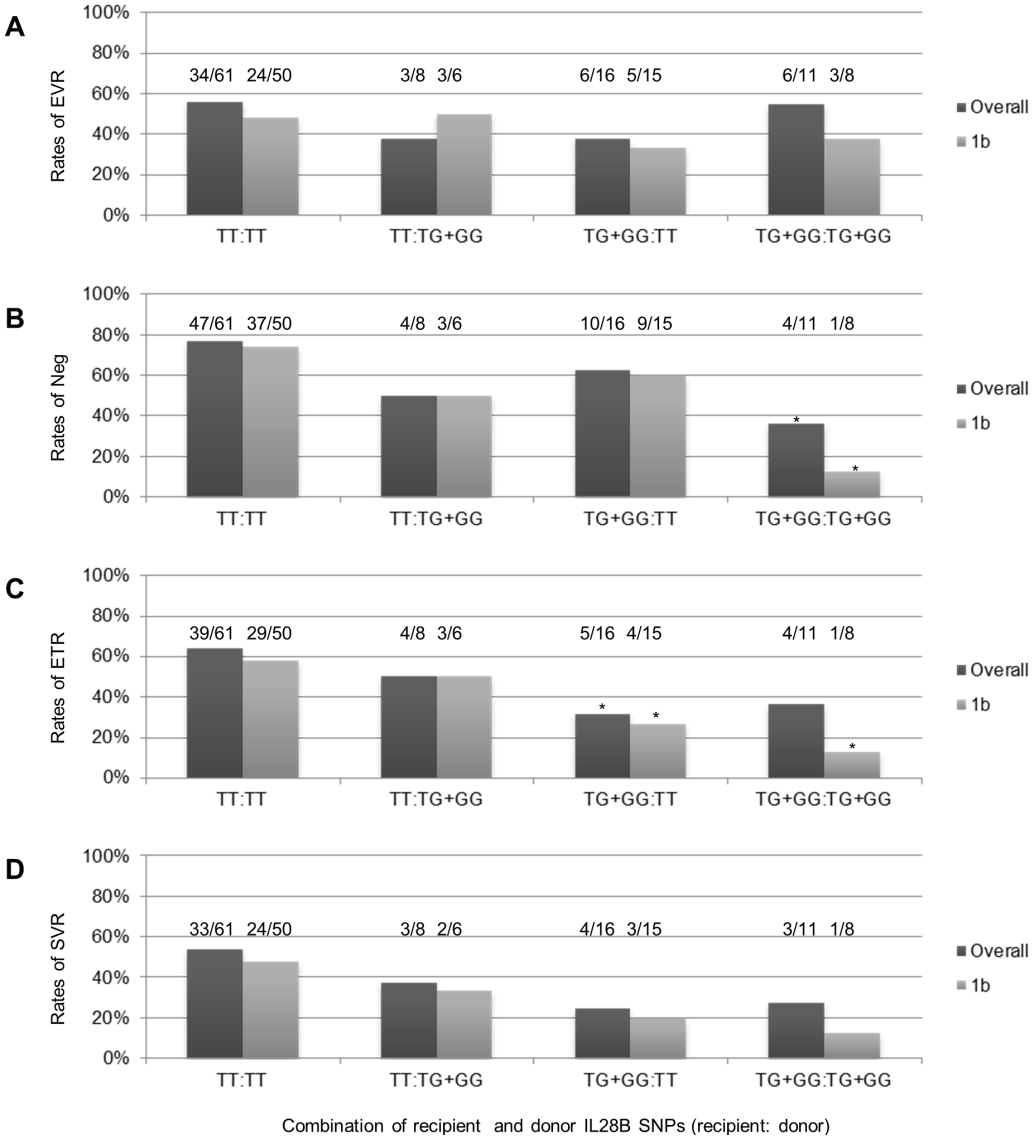
Combination of recipient and donor IL28B rs8099917 SNPs and corresponding rates of viral responses (A–D). A. Rates of EVR, B. Rates of recipients that presented with temporal non-detectable level of serum HCV RNA at least once during the course of INF treatment, C. rates of ETR, D. rates of SVR. Abbreviation: SNPs, single nucleotide polymorphism; SVR, sustained viral response; EVR, early virologic response; Neg, temporal non-detectable level of serum HCV RNA; ETR, end of treatment response. * indicates p = <0.05 v.s. combination of major allele both in the recipient and donor (TT:TT) with corresponding background.

The combinations of recipient and donor IL28B polymorphisms and corresponding rates of virologic responses are summarized in Figures 2A–2D. Although combinations of recipients and grafts obtained from donors both carrying the major homozygous allele presented with tendency of higher rates of virologic eradication demonstrated as ETR (Figure 2C) or SVR (Figure 2D), especially in the case of HCV RNA genotype 1b, this synergistic tendency remained unclear or limited when observation was extended to on-treatment virologic response at an earlier stage evaluated by temporal clearance or EVR (Figures 2A and 2B). For example, rates of EVR between recipient and donor pairs carrying both a major homozygous allele and minor allele did not differ significantly (p = 0.9416), and the advantage remained unclear even when limited to the HCV RNA genotype 1b (p = 0.5804) (Figure 2A). Similarly, the presence of a minor allele either in the recipient or the donor did not significantly affect temporal viral clearance (Figure 2B).

### Impact of IL28B polymorphism among other factors

**Figure 3:**
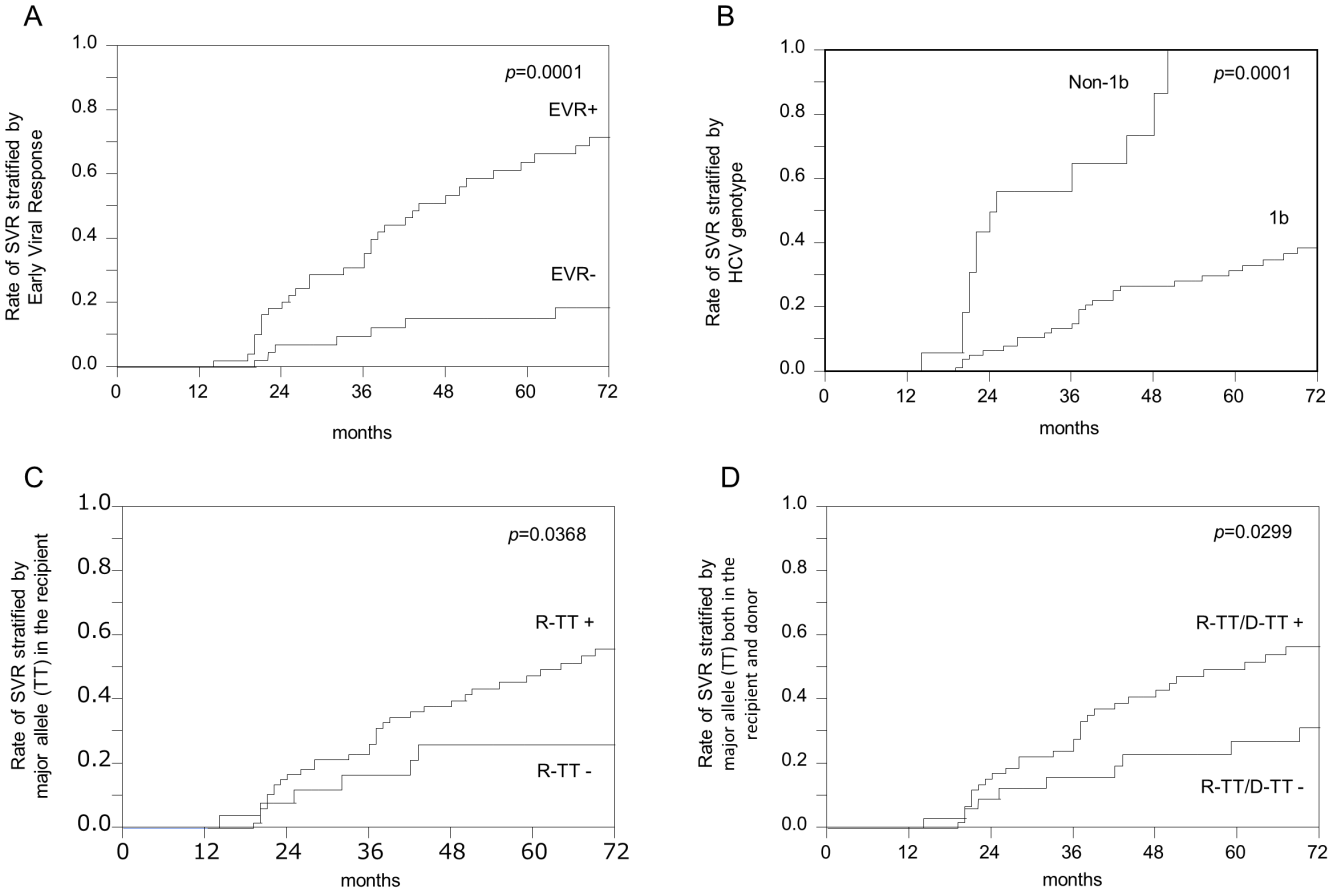
Cumulative rates of SVR based on the Kaplan-Meier method. A. Stratified by EVR, B. HCV genotype, C. major allele (TT) in the recipient, and D. major allele (TT) both in the recipient and donor. Abbreviations: SVR, sustained viral response; EVR, Early viral response; R-TT, major allele (TT) in the recipient; D-TT, major allele (TT) in the donor.

Impact of various clinical factors on sensitivity to interferon treatment in the present study was assessed by SVR rates (Table 1). Compatible with previous studies, univariate analysis revealed that HCV genotype 1b and presence of EVR were significant factors affecting outcome. As for IL28B polymorphisms, a major allele homozygote in the recipient, and a major allele homozygote both in the recipient and donor presented with a statistically significant impact (p = 0.0368, and 0.0299, respectively). A major allele homozygote in the donor side alone did not have strong impact (0.3136; Table 1). The above four factors significantly impacted SVR in univariate analysis are summarized in Figure 3.

**Table 1:** Sustained viral response in patients with combined treatment and clinical factors.

Factors		No.	%SVR at 5 y	p
R-age	= <60	72	48	
	>60	24	26	0.5221
R-sex	Male	70	44	
	Female	26	34	0.5895
MELD	= <15	52	42	
	>15	44	40	0.9585
HIV	Positive	3	67	
	Negative	93	40	0.0729
HCC	Positive	60	41	
	Negative	36	48	0.7344
Genotype	1b	79	32	
	Non-1b	17	100	<0.0001
Graft size	<50%	53	46	
%R-SLV	> = 50%	43	37	0.8251
HCV-RNA	= <5.6	48	45	
titer	>5.6	48	38	0.2999
EVR	Yes	49	64	
	No	47	15	<0.0001
ACR	Yes	20	49	
	No	76	39	0.3844
D-age	= <40	61	39	
	>40	35	46	0.1101
D-sex	Male	62	33	
	Female	34	59	0.1155
CyA	Yes	58	51	
	No	38	27	0.0683
R-IL28B	TT	69	47	
	TG/GG	27	26	0.0368
D-IL28B	TT	77	44	
	TG/GG	19	31	0.3136
R-TT/D-TT	Yes	61	49	
	No	35	27	0.0299
R-TT/D-TG+GG	Yes	8	31	
	No	88	42	0.7276
R-TG+GG/D-TT	Yes	16	22	
	No	80	45	0.0962
R-TG+GG/D-TG+GG	Yes	11	31	
	No	85	43	0.3386

Abbreviations: No., number of patients; %SVR, rate of recipients achieving sustained viral response; R-age, age of the recipient at the time of transplantation; R-sex, sex of the recipient; MELD, Model for end-stage liver disease score; HIV, human immune deficiency virus; HCC, hepatocellular carcinoma; HCV, hepatitis C virus; %R-SLV, percentage of graft size to recipient's standard liver volume; HCV-RNA, hepatitis C viral ribonucleic acid; EVR, early viral response; ACR, acute cellular rejection; D-age, age of the donor at the time of transplantation; D-sex, sex of the donor; CyA, cyclosporine A; R-IL28B, recipient's IL28B polymorphism (rs8099917); D-IL28B, donor's IL28B polymorphism (rs8099917); R-TT/D-TT, Both recipient and donor carrying major allele (TT); R-TT/D-TG+GG, recipient carrying major allele (TT) but donor carrying minor allele (TG or GG); R-TG+GG/D-TT, recipient carrying minor allele (TG or GG) but donor carrying major allele (TT); R-TG+GG/D-TG+GG, Both recipient and donor carrying minor allele (TG or GG).

**Figure 4:**
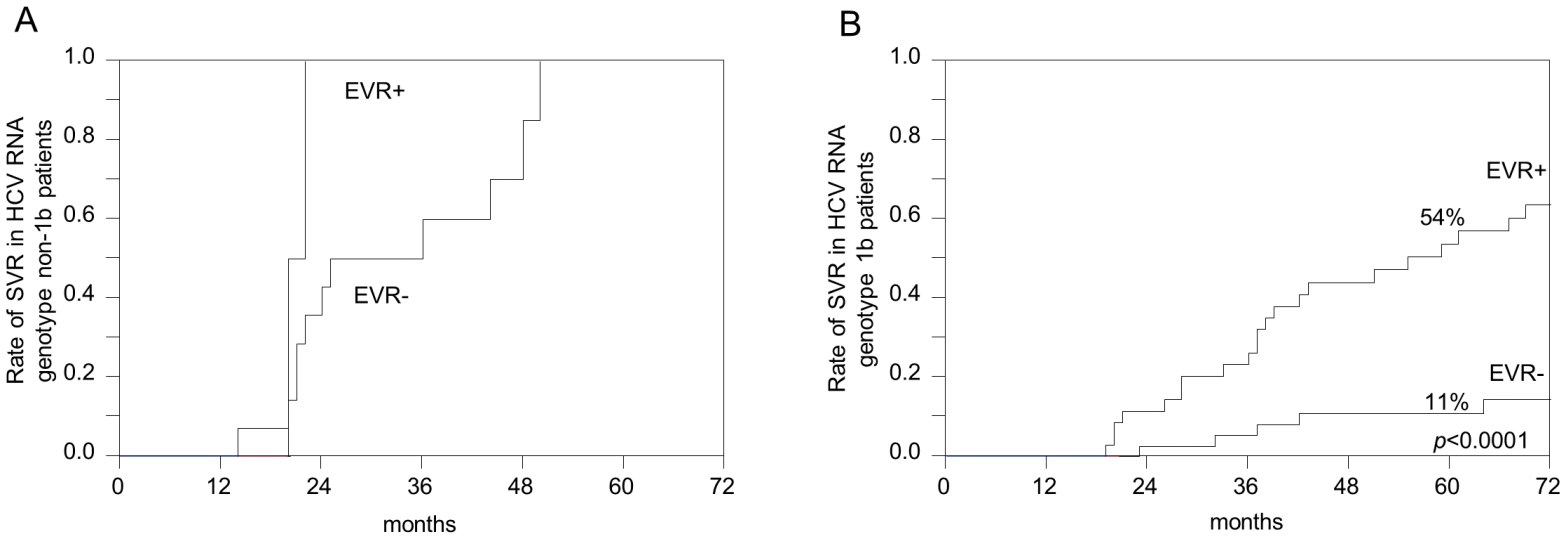
Cumulative rates of SVR stratified by EVR using the Kaplan-Meier method. A. HCV RNA genotype non-1b, B. HCV RNA genotype 1b. Abbreviations: SVR, sustained viral response; EVR, Early viral response.

To elucidate the magnitude of the IL28B polymorphism, a multivariate study was conducted including all clinical variables from Table 1. To incorporate the nature of our treatment protocol without a defined period of interferon treatment, multivariate analysis was performed using the Cox proportional-hazards model. The study revealed the genotype of HCV and EVR, but not the IL28B polymorphism of either recipient or donor, to be independent factors to achieve SVR. Recipients re-infected with HCV genotype 1b presented with a significantly poorer chance of SVR (Hazard ratio 0.277, 95% confidence interval 0.132–582, p = 0.0007). On the other hand, once EVR was observed, recipients demonstrated significantly better opportunity for SVR (Hazard ratio 4.426, 95% confidence interval 1.958–10.007, p = 0.0004). Cumulative rates of SVR within the genotype 1b or non-1b population stratified by the presence of EVR are presented in Figure 4. Conversely, background factors of recipients presenting EVR or not so depending on HCV RNA genotypes were evaluated (Table 2). Among various clinical factors, IL28B polymorphisms, either that of the recipient or the donor, or both, was not prevalent in relation to EVR, especially in the genotype 1b population.

**Table 2:** Early viral response in patients with combined treatment and clinical factors.

		HCV RNA Genotype					
		Non-1b			1b		
Factors		EVR		p	EVR		p
		No	Yes		No	Yes	
R-age	= <60	2	11		31	28	
	>60	1	3	1.0000	13	7	0.4368
R-sex	Male	2	10		33	25	
	Female	1	4	1.0000	11	10	0.8001
MELD	= <15	3	5		26	18	
	>15	0	9	0.0824	18	17	0.6488
HIV	Positive	0	3		0	0	
	Negative	3	11	1.0000	44	35	NA
HCC	Positive	2	5		28	25	
	Negative	1	9	0.5368	16	10	0.4826
Graft size	<50%	1	7		26	19	
%R-SLV	> = 50%	2	7	1.0000	18	16	0.8194
HCV-RNA titer	= <5.6	3	8		24	13	
	>5.6	0	6	0.5147	20	22	0.1735
ACR	Yes	0	2		7	11	
	No	3	12	1.0000	37	24	0.1149
D-age	= <40	1	7		28	25	
	>40	2	7	1.0000	16	10	0.4826
D-sex	Male	2	8		27	25	
	Female	1	6	0.6704	17	10	0.4744
CyA	Yes	0	1		19	18	
	No	3	13	1.0000	25	17	0.5029
R-IL28B	TT	3	10		29	27	
	TG/GG	0	4	0.5412	15	8	0.3251
D-IL28B	TT	1	11		36	29	
	TG/GG	2	3	0.1912	8	6	1.0000
R-TT/D-TT	Yes	1	10		26	24	
	No	2	4	0.5147	18	11	0.4826
R-TT/D-TG+GG	Yes	2	0		3	3	
	No	1	14	0.0221	41	32	1.0000
R-TG+GG/D-TT	Yes	0	1		10	5	
	No	3	13	1.0000	34	30	0.3983
R-TG+GG/D-TG+GG	Yes	0	3		5	3	
	No	3	11	1.0000	39	32	1.0000

Abbreviations: HCV-RNA, hepatitis C viral ribonucleic acid; EVR, early viral response; R-age, age of the recipient at the time of transplantation; R-sex, sex of the recipient; MELD, Model for end-stage liver disease score; HIV, human immune deficiency virus; HCC, hepatocellular carcinoma; HCV, hepatitis C virus; %R-SLV, percentage of graft size to recipient's standard liver volume; ACR, acute cellular rejection; D-age, age of the donor at the time of transplantation; D-sex, sex of the donor; CyA, cyclosporine A; R-IL28B, recipient's IL28B polymorphism (rs8099917); D-IL28B, donor's IL28B polymorphism (rs8099917); R-TT/D-TT, Both recipient and donor carrying major allele (TT); R-TT/D-TG+GG, recipient carrying major allele (TT) but donor carrying minor allele (TG or GG); R-TG+GG/D-TT, recipient carrying minor allele (TG or GG) but donor carrying major allele (TT); R-TG+GG/D-TG+GG, Both recipient and donor carrying minor allele (TG or GG).

Overall, IL28B polymorphisms had a relative, not independent, impact. On-treatment virologic response to interferon and RBV-based treatment represented by EVR remain the most significant factor predicting SVR, even among recipients with HCV genotype 1b.

## Discussion

Treatment of HCV re-infection by interferon and ribavirin after liver transplantation has remained a challenge with inferior outcomes compared to the non-transplantation population due to immunosuppression, and low tolerability. Recurrence and persistence of HCV-infection remain the most common cause of post-transplant graft loss and mortality. Identifying factors affecting the outcomes, including the response to treatment, therefore, continues to be a subject of keen interest. This study presents observations that may be potentially important in light of advancements involving recent genetic discoveries regarding IL28B polymorphisms.

This is the largest study to date, 96 LDLT cases, evaluating the impact of IL28B polymorphisms in both donor and recipient, in accordance with the on-treatment response, on the outcome. Also, the series is the first to analyze the magnitude of the polymorphism under a preemptive treatment approach [17], [18] after LDLT. The study is limited to rs8099917 based on previous studies of IL28B SNPs in the Japanese population, and therefore, impact of rs12979860 awaits further similar study in the West.

Several predictive factors for interferon and RBV sensitivity in the non-transplant population were recently identified. Virologically, HCV-genotype, and HCV RNA mutations in the core and NS5A regions are recognized as important factors [24], [25]. As for host factors, by genome-wide association study coming from three independent studies, IL28B polymorphisms have been identified as significant factors affecting virologic clearance [19]–[21]. The mechanism underlying the influence of IL28B polymorphisms on the response to interferon and RBV therapy is, however, yet to be determined. Current understanding is that the product of the IL28B gene is interferon lambda-3, which belongs to the type III interferon family that induces interferon-stimulated genes. Favorable IL28B polymorphisms are associated with decreased levels of intrahepatic interferon-stimulated genes, offering a favorable environment for virologic clearance under interferon and RBV treatment [26]–[28]. Whether or not this mechanism is applicable to the liver transplant setting, in which a liver allograft is infected by HCV under immunosuppression, remains to be studied. There is little evidence to speculate otherwise at this point.

Fukuhara and colleagues [22] first reported the impact of IL28B polymorphisms on the outcome of LDLT. In their study, IL28B polymorphisms were studied in 67 HCV infected recipients and 41 living liver donors. Interestingly, they reported that SVR achievement was significantly associated with IL28B polymorphisms of both the recipient and donor. When both were major-allele homozygotes, the SVR rate was 56%. Whereas when either the recipient or the donor presented with a minor heterozygote or homozygote allele, SVR rate dropped to 10%, and further, when both the recipient and donor presented with a minor heterozygote or homozygote allele, none achieved SVR. Kawaoka and colleagues [23] conducted a similar study involving 20 LDLT recipient and donor pairs. They reported that major-allele homozygotes in both the recipient and donor resulted in an SVR rate of 81%. Although the number of cases where rather small, multivariate analysis including adherence to RBV therapy was performed, revealing that major-allele homozygotes in both the recipient and donor as the only independent and dominant determinant of SVR with and an odds ratio of 15.

Although logistic and technical difficulties remain in sampling and analyzing IL28B gene of the donors, comparable studies have been performed in the deceased donor setting [29]–[34]. The impact of IL28B polymorphisms has also become recognized in deceased donor liver transplantation for HCV. It was suggested that, patients requiring liver transplantation due to end-stage chronic HCV appeared to be selected toward the adverse genotypes [31], and the polymorphism seems to influence the degree of graft inflammation at biochemical and histologic levels following transplantation [29], [32]. It has also become evident that, while there seems to be little doubt that IL28B polymorphisms markedly affect the response to interferon and RBV treatment, whether the donor or recipient, or the combination of both, should be considered paramount differ among studies. Lange and colleagues provide evidence that the donor's rather than the recipient's IL28B genetic background has a dominant impact on the virologic response [31], while Cotpo-Llerena et al. [34] report that the recipient's genetic background plays a major role. On the other hand, a recent study by Duarte-Rojo and colleagues [30] used multivariate analysis to demonstrate that the combination of both is the most influential. A German study [32] provided no data on the potential role of donor IL28B polymorphisms. The numbers of subjects in these studies remain small in comparison with the size of patients involved in analysis of non-transplant cases. Data on previously reported important clinical factors other than IL28B polymorphisms are not readily available for evaluation; much less an analysis by multivariate analysis to weigh the impact in a more reliable context. Clearly, further studies are required with an inclusion of a broader range of clinical data.

In our study, we included factors previously reported to influence the outcomes of the virologic response against interferon and RBV therapy in the analysis. This includes age and sex of both the donor and recipient, preoperative viral load, immunosuppression, and other factors as well as the on-treatment results represented by EVR (Table 1). A recent report by Thompson and colleagues suggests that on-treatment virologic response may have strong predictive power regardless of the IL28B type [35]. We used multivariate analysis that included all of these factors, and found that IL28B polymorphism is an influential but not determinant factor for SVR. Rather, in our series with a preemptive treatment approach, we demonstrated that on-treatment response was the key factor for predicting SVR.

Our study has three major weaknesses. First, although clinical virologic response was followed up and recorded in a prospective manner, IL28B polymorphisms were recently determined, making our study a retrospective case series with diverse sources of DNA. DNA samples for analysis were collected either from peripheral blood mononuclear cells or from formalin-fixed paraffin-embedded samples based on availability. A prospective study with a fixed DNA sampling protocol is required. Second, the nucleotide sequences of the core and non-structural 5A regions, another recently suggested important factor [36], have not been investigated in concert with IL28B polymorphisms. In fact, few studies to date have performed a combined analysis of both IL28B polymorphisms and HCV RNA nucleotide sequences, most likely due to the additional logistic burden. Fukuhara and colleagues [22] reported the synergistic value of combining findings from IL28B polymorphisms and HCV RNA nucleotide sequences in predicting the treatment response. This aspect should also be considered in future studies. Third, the study lacks histologic data. In our series, protocol biopsy was not performed. Hepatic venous gradient to evaluate the degree of liver fibrosis was also not routinely performed. This is due in part to our preemptive treatment strategy. Eurich and colleagues [32] have presented interesting outcomes regarding the progression of the histologic response in their deceased donor series. Comparable analysis in the living donor setting in the future may be valuable.

Finally, in the current study, direct antiviral agents in combination with peg-IFN and ribavirin were not used. Although the efficacy of the earlier generation of direct antiviral agents has become recognized in the non-transplant population, drastically altering standard treatment [37]–[39], its safety and effectiveness under routine use in the transplant population await future confirmation. Development in this aspect, however, is in rapid progression. Current recognition is that new-age anti-HCV treatment incorporating advanced direct antiviral agents will radically alter the outcome [40]. Further accumulation of data in combination with IL28B and the development of additional treatment options may be beneficial.

### Conclusions

In contrast to previous reports, when virologic response to treatment was incorporated into analysis, the impact of IL28B polymorphism on achieving SVR remained relative in our living donor liver transplantation series under a preemptive interferon and RBV-based treatment approach. HCV genotype 1b and on-treatment response represented by EVR were both significant and independent factors. Caution should be used when incorporating the IL28B polymorphism into the treatment strategy of HCV reinfection following liver transplantation in an absolute manner, such as to the donor selection or graft allocation, however, until the mechanism of its effect is elucidated and well-designed future studies have confirmed its true nature.
